# A database for the taxonomic and phylogenetic identification of the genus *Bradyrhizobium *using multilocus sequence analysis

**DOI:** 10.1186/1471-2164-16-S5-S10

**Published:** 2015-05-26

**Authors:** Helton Azevedo, Fabricio Martins Lopes, Paulo Roberto Silla, Mariangela Hungria

**Affiliations:** 1 Federal University of Technology - Paraná, Av. Alberto Carazzai, 1640, 86300-000 Cornélio Procópio, Brazil; 2 Empresa Brasileira de Pesquisa Agropecuária - Embrapa, João Carlos Strass, Londrina, Brazil

**Keywords:** *Bradyrhizobium *database, Taxonomic of Prokaryotes, Phylogeny of Prokaryotes, Multilocus Sequence Analysis, 16S rRNA Gene, Bioinformatics, Pattern Recognition

## Abstract

**Background:**

Biological nitrogen fixation, with an emphasis on the legume-rhizobia symbiosis, is a key process for agriculture and the environment, allowing the replacement of nitrogen fertilizers, reducing water pollution by nitrate as well as emission of greenhouse gases. Soils contain numerous strains belonging to the bacterial genus *Bradyrhizobium*, which establish symbioses with a variety of legumes. However, due to the high conservation of *Bradyrhizobium *16S rRNA genes - considered as the backbone of the taxonomy of prokaryotes - few species have been delineated. The multilocus sequence analysis (MLSA) methodology, which includes analysis of housekeeping genes, has been shown to be promising and powerful for defining bacterial species, and, in this study, it was applied to *Bradyrhizobium*, species, increasing our understanding of the diversity of nitrogen-fixing bacteria.

**Description:**

Classification of bacteria of agronomic importance is relevant to biodiversity, as well as to biotechnological manipulation to improve agricultural productivity. We propose the construction of an online database that will provide information and tools using MLSA to improve phylogenetic and taxonomic characterization of *Bradyrhizobium*, allowing the comparison of genomic sequences with those of type and representative strains of each species.

**Conclusion:**

A database for the taxonomic and phylogenetic identification of the *Bradyrhizobium*, genus, using MLSA, will facilitate the use of biological data available through an intuitive web interface. Sequences stored in the on-line database can be compared with multiple sequences of other strains with simplicity and agility through multiple alignment algorithms and computational routines integrated into the database. The proposed database and software tools are available at http://mlsa.cnpso.embrapa.br, and can be used, free of charge, by researchers worldwide to classify *Bradyrhizobium*, strains; the database and software can be applied to replicate the experiments presented in this study as well as to generate new experiments. The next step will be expansion of the database to include other rhizobial species.

## Background

Taxonomy of prokaryotes is gaining increasing attention duo to both the valoration of biodiversity and the recognition of the economic value of many microorganisms. Phylogenetic studies are also key for determining the exact taxonomic position of organisms, as well as to determine their evolutionary history, indicating their relations with other groups and their places in families and kingdoms.

Bacterial phylogeny is based mainly on sequence data of biological macro-molecules; highly conserved molecules help to compare distantly related organisms, whereas molecules that change rapidly help to elucidate small and recent changes [1]. The 16S rRNA gene is broadly elected as the backbone of prokaryote taxonomy and phylogeny [2] and repositories of both 16S rRNA genes and other biological data are increasing every day, generating large datasets [3]; efficient organization of this information is critical to scientific progress.

The term "rhizobia" applies to soil-borne bacteria that are capable of fixing atmospheric nitrogen *N*_2 _in symbioses with, and for the benefit of, plants, the vast majority of which are legumes. Yearly, billions of dollars are saved worldwide thanks to the action of rhizobia, in crops that otherwise would require application of nitrogen fertilizers to achieve optimal yields. However, despite their importance to the agriculture and to the environment, studies on phylogeny and taxonomy of rhizobia are relatively scarce, including in some countries where genetic diversity is high, such as Brazil [4]. The genus *Bradyrhizobium*, used in this study, is currently composed of 19 species recognized by the International Committee of Taxonomy; it has been suggested to be the ancestor of all rhizobia, having originated in the tropics e.g. [5-8]. The genus includes important strains, such as those known to contribute superior rates of *N*_2 _fixation to grain crops such as soybean (*Glycine max (L.) Merr.*) [9]. However, one main limitation in taxonomy and phylogeny studies of *Bradyrhizobium *is that its 16S rRNA gene is highly conserved, making it difficult to capture the diversity observed in other phenotypic and genotypic analyses and to define and delineate species [4,10-13]. Therefore, one interesting approach has been to use the multilocus sequencing analysis (MLSA) methodology, including the analysis of housekeeping genes which is conserved but with a higher rate of evolution, to more precisely detect diversity within the genus *Bradyrhizobium *[8,12,14].

Some technologies have been developed in order to improve the identification process of biological entities, such as PseudoMLSA Database [15] and EZTaxon [16]. The former has a model similar to that proposed in our study, including the possibility of performing similarity searches using Blast [17], phylogenetic inference by CLUSTAL Omega [18] and PHYLIP [19] for *Pseudomonas *species. With EZTaxon [16] it is possible to identify all types of prokaryotes, using an information database along with 16S rRNA gene sequences. By contrast, our study provides a new database with the combination of different software tools for multiple sequence alignments and techniques for automatic pre-processing and post-processing the genomic sequences that are necessary for carrying out the MLSA, and, hence, identify biological entities.

The database for taxonomic identification and phylogenetics of the genus *Bradyrhizobium *through MLSA described in our study represents a repository for genomic sequences of *Bradyrhizobium *species. The main objective is to be an online database, open sourced with helpful information and tools in order to elucidate the taxonomy and phylogenetic analysis of these organisms. The current version of the database represents a selection of genes assigned to the genus *Bradyrhizobium *that are commonly used and are validated, and were updated through June 2014. The web interface developed for this system enables users to perform analyses of similarity of their datasets, as well as to make queries and downloads in the stored genomic sequences.

The need for a more informative database of species of rhizobia with useful genes for applying the MLSA methodology results from the fact that currently generated sequences for identification and rating of these organisms are scattered across various databases, and gathering this information is a time-consuming process. We started the procedure with the genus *Bradyrhizobium* - i.e. the most difficult in terms of rhizobial taxonomy - due to its highly conserved 16S rRNA gene sequence [9-14] and due to interest in its evolution since it is considered as the ancestor of all rhizobia [5-9]. In due course, the database will be expanded to include other rhizobial species.

### Current Taxonomic Analysis

Taxonomic consensus is best achieved when different types of data and information (phenotypic, genotypic, phylogenetic) are combined. This integrated model of information is called polyphasic taxonomy, and a bacterial species is defined as a group of genomically alike strains that share a high degree of similarity in several independent features [20]. The phenotypic data are obtained through studies involving gene expression, protein analysis and function, chemotaxonomic markers, and other characteristics that correspond to the final expression of genes [21-23]. For genotyping studies, the information is obtained from both DNA and RNA. Various methodologies can be cited for this purpose, including G+C mol% of DNA; DNA-DNA hybridization (DDH); restriction-fragment-length polymorphism (RFLP); pulsed-field gel electrophoresis (PFGE); gene sequencing; and PCR-fingerprinting [24]. The DDH method is based on physico-chemical properties of the DNA and has been required for the definition of most prokaryote species. However, DDH has several limitations, including low reproducibility among laboratories, high labour demand, cost and time consumption due to the need for hybridization of a large number of strains [23,25]. Furthermore, there is no database that allows the comparison of results from different studies [26].

Comparisons of the ribosomal 16S rRNA gene represent the basis of modern taxonomic analysis; important databases comprise 16S rRNA genes, such as the ribosomal database project at https://rdp.cme.msu.edu. However, a limitation is the high degree of nucleotide-sequence conservation in this gene across genera-including *Bradyrhizobium*-makiiig it difficult to distinguish closely related species [24,27-32]. Consequently, it is important to develop new techniques that can complement the results obtained from 16S rRNA gene-sequence data, as well as replace DDH for taxonomic purposes. It is also important to establish databases that facilitate analyses of new strains.

### Multilocus Sequence Analysis (MLSA)

Identifying organisms as prokaryotic and the delineation of species are the main foci of the taxonomy of microorganisms [33]. Thus, although the levels of identity-obtained in the analysis of the sequences of the 16S rRNA gene and of DDH are still considered as molecular criteria for classification of species, it is expected that additional taxonomic information can be obtained from complete genome sequences [34], and MLSA has been increasingly suggested as a replacement for DDH [9,35,36].

MLSA represents a strategic alternative to avoid the effects of genetic recombination and horizontal transfer occurring in a specific single gene [33,35]. In addition, it can clarify the distinction between highly related species, or species where the analysis of the 16S rRNA genes shows low resolution, since the chosen housekeeping genes-comprising genes involved in cellular metabolism, i.e. those essential for the survival of the microorganism [35]-present faster evolutionary rates than do the ribosomal genes, but with a level of conservation sufficient to reveal evolutionary information [21,24,25,27,36]. The choice of housekeeping genes should follow certain criteria, including: i) presence in the genome in a single copy; ii) being distributed in the genome with a minimal distance between the genes of 100 kb; iii) containing sufficient nucleotide length to allow its sequencing; iv) containing sufficient information for its analysis [13,25,27,36-38] .

The MLSA methodology has been increasingly used to improve bacterial taxonomy, providing a tool suitable for defining species and revealing their taxonomic relationships. Several studies have shown that MLSA may provide high resolution, allowing the discrimination of isolates at the species level [14,25,36,38-41], which would not be possible by analysis exclusively by 16S rRNA-gene sequencing [12,33,35]. The distinction at the species level is achieved by MLSA analysis through algorithms for estimating evolutionary distance between strains. In the particular case of rhizobia, housekeeping genes used in recent years as phylogenetic markers for the species classification include atpD, recA, glnA, glnB, dnaK, thrC and git A [4]. However, taking into account the large number of microorganisms that remain to be identified and classified, and the improvement of microbiology data generation, there is need for the development of new databases and software tools for their analysis [33,35].

## Construction and content

The computational infrastructure used to provide the set of services described in this work is hosted at the National Soybean Research Center of the Brazilian Agricultural Research Corporation (Embrapa Soja). All applications and tools required for the operation of the database were configured for the platform Linux Ubuntu Server 4.13 with Apache 2.4.7, the MySQL database-management system, and the phpAdmin 4.2.2 data-modelling tool.

The relational model of the proposed database follows the scheme proposed by the BioSQL project [42], considering that it is a standard solution for storing sequences of molecular modelling, and it has compatibility with other bioinformatics projects such as BioPerl, BioPython, BioJava and BioRuby. The database was developed by considering the same data structure used in GenBank [43]. Therefore, it is expected that the database-updating process will not be a time-consuming task, and its usability can be improved in the future. BioSQL allows customization of its schema through extension modules, such as the PhyloDB, which allows the storage of taxonomy and phylogenetic trees. Besides MySQL, relational databases such as PostgreSQL, HSQLDB, Apache Derby and Oracle also support this bioinformatics tool. The adopted BioSQL schema is available as additional file 1.

GenBank files are used to provide the required information and keep it updated in the database. Sequences, resources and notes are included in the database from BioPython scripts and the SeqIO module [44]. Multiple alignments were adopted by means of the algorithms CLUSTAL Omega [18] and MUSCLE [45]. The verification of the homology between nucleotides of the bacterial genes was also integrated as a software tool into the web interface of the proposed database. This process is very important for identifying regions aligned among various species and plays a key role in the application of the MLSA methodology, in order that only after aligning and trimming of all the analysed sequences of equal size, it is possible to perform the phylogenetic and taxonomic inferences of the analysed species. The multiple sequence alignment is performed by means of web services developed by the European Bioinformatics Institute (EMBL-EBI), available for CLUSTAL Omega [http://www.ebi.ac.uk/Tools/webservices/services/msa/clustalo_soap] and for MUSCLE [http://www.ebi.ac.uk/Tools/webservices/services/msa/muscle_soap].

Finally, scripts in PHP and Java Script were developed in order to parameterize and to perform the post processing of the bioinformatics tools available in the database. These scripts are important to make the cropping areas of common genes aligned, allowing individual analyses of these genes and concatenating the loci for the application of the MLSA methodology.

The database presented in this work consists of 286 genomic sequences, distributed in six specific housekeeping genes, namely: atpD, dnaK, glnll, recA, gyrB and rpoB. Nineteen species of the Bradyrhizobium genus were considered: *B. betae, B. canariense, B. cytsi, B. daqingense, B. denitrificans, B. diazoefficiens, B. elkanii, B. huanghuaihaiense, B. icense, B. iriomotense, B. japonicum, B. jicamae, B. lablabi, B. liaoningense, B. oligorophicum, B. pachyrhizi, B. paxllaeri, B. rifense and B. yuanmingense*.

For species such as *B. canariense, B. diazoefficiens, B. elkani, B. japonicum, B. liaoningense *and *B. yuanmingense *other reference strains were included in order to improve the molecular and phylogenetic characterizations and to refine the process of comparison of results. Accession numbers of the sequences used in this work are available in Table 1 and for building the phylogenetic trees, the species *Rhodopseudomonas palustris *was adopted as an outgroup.

**Table 1:** GenBank accession numbers of the sequences used in this work.

Strain	Genome	atpD	dnaK	glnll	recA	gyrB	rpoB
*B. betae *LMG 21987*^T^*		FM253129.1	AY923046.1	AB353733.1	AB353734.1	FM253217.1	FM253260.1
*B. canariense *LMG 22265*^T^*		AY386739.1	AY923047.1	AY386765.1	FM253177.1	FM253220.1	FM253263.1
*B. cylisiCTAW *11*^T^*		GU001613.1	KF532219.1	GU001594.1	GU001575.1	KF532653.1	JN186288.1
*B. dagingense *CCBAU 15774*^T^*		HQ231289.1	KF962684.1	HQ231301.1	HQ231270.1	KF962694.1	JX437676.1
B. deniirificans 8443		FM253153.1	KF962685.1	HM047121.1	FM253196.1	FM253239.1	FM253282.1
*B. diazoefficiens *USDA 110*^T^*	NC 004463.1	NC 004463.1	NC 004463.1	NC 004463.1	NC 004463.1	NC 004463.1	NC 004463.1
*B. elkanii *USDA 76*^T^*		AY386758.1	AY328392.1	AY599117.1	AY591568.1	AM418800.1	AM 295348.1
*B. huanghuaihaiense *CCBAU 23303*^T^*		HQ231682.1	KF962686.1	HQ231639.1	HQ231595.1	KF962695.1	HQ428068.1
*B. iriomoiense EK *05*^T^*		AB300994.1	JF308944.1	AB300995.1	AB300996.1	AB300997.1	HQ587646.1
*B. japonicum *USDA 6*^T^*		AM168320.1	AM168362.1	AF169582.1	AM182158.1	AM418801.1	AM295349.1
*B. jicamae *PAC 68*^T^*		FJ428211.1	JF308945.1	FJ428204.1	HM047133.1	HQ873309.1	HQ587647.1
*B. lablabi *CCBAU 23086*^T^*		GU433473.1	KF962687.1	GU433498.1	GU433522.1	KF962696.1	JX437677.1
*B. liaoningense *LMG 18230*^T^*		AY386752.1	AY923041.1	AY386775.1	AY591564.1	FM253223.1	FM253266.1
*B. pachyrhizi *PAC 48*^T^*		FJ428208.1	JF308946.1	FJ428201.1	HM047130.1	HQ873310.1	HQ587648.1
*B. rifense CTAW *71*^T^*		GU001617.1	KF532220.1	GU001604.1	GU001585.1	KF532666.1	KC569468.1
*B. yuanmingense *LMG 21827*^T^*		AY386760.1	AY923039.1	AY386780.1	AM168343.1	FM253226.1	FM253269.1
*B. icense *LMTR 13		KF896192.1	KF896182.1	KF896175.1	JX943615.1	KF896201.1	
*B. oligoirophicum *LMG 10732		JQ619232.1	KF962688.1	JQ619233.1	JQ619231.1	KF962697.1	KF962713.1
*B. paxllaeri *LMTR 21		KF896186.1	AY923038.1	KF896169.1	JX943617.1	KF896195.1	
*Rhodopseudomonas palusiris *CGA009	NC 005296.1	NC 005296.1	NC 005296.1	NC 005296.1	NC 005296.1	NC 005296.1	NC 005296.1
SEMIA 5025		FJ390951	FJ390991	FJ391031	FJ391151		
SEMIA 5045		FJ390954	FJ390994	FJ391034	FJ391154		
SEMIA 5060		JX867237.1	JX867240.1	JX867241.1	JX867239.1	JX867245.1	JX867242.1
SEMIA 5062		FJ390955	FJ390995	FJ391035	FJ391155		
SEMIA 5079	CP007569.1	FJ390956.1	FJ390996.1	FJ391036.1	FJ391156.1	CP007569	CP007569
SEMIA 5080		FJ390957.1	FJ390997.1	FJ391037.1	FJ391157.1	JX867246.1	JX867243.1
SEMIA 511		FJ390942	FJ390982	FJ391022	FJ391142		
SEMIA 512		FJ390943	FJ390983	FJ391023	FJ391143		
SEMIA 560		FJ390944	FJ390984	FJ391024	FJ391144		
SEMIA 6014		FJ390958	FJ390998	FJ391038	FJ391158		
SEMIA 6028		FJ390959	FJ390999	FJ391039	FJ391159	HQ634886	HQ634905
SEMIA 6053		FJ390960	FJ391000	FJ391040	FJ391160	HQ634887	HQ634906
SEMIA 6059		FJ390961.1	FJ391001.1	FJ391041.1	FJ391161.1	JX867247.1	JX867244.1
SEMIA 6069		FJ390962	FJ391002	FJ391042	FJ391162		
SEMIA 6077		FJ390963	FJ391003	FJ391043	FJ391163		
SEMIA 6093		FJ390964	FJ391004	FJ391044	FJ391164		
SEMIA 6099		FJ390965	FJ391005	FJ391045	FJ391165		
SEMIA 6101		FJ390966	FJ391006	FJ391046	FJ391166		
SEMIA 6144		HQ634873	EU196049	HQ634879	HQ634897	HQ634888	HQ634907
SEMIA 6146		FJ390967	FJ391007	FJ391047	FJ391167		
SEMIA 6148		FJ390968	FJ391008	FJ391048	FJ391168	HQ634890	HQ634909
SEMIA 6152		FJ390969	FJ391009	FJ391049	FJ391169		
SEMIA 6156		FJ390970	FJ391010	FJ391050	FJ391170		
SEMIA 6160		FJ390971	FJ391011	FJ391051	FJ391171	HQ634892	HQ634911
SEMIA 6163		FJ390972	FJ391012	FJ391052	FJ391172		
SEMIA 6164		FJ390973	FJ391013	FJ391053	FJ391173		
SEMIA 6179		FJ390974	FJ391014	FJ391054	FJ391174		
SEMIA 6186		FJ390975	FJ391015	FJ391055	FJ391175		
SEMIA 6187		FJ390976	FJ391016	FJ391056	FJ391176		
SEMIA 6192		FJ390977	FJ391017	FJ391057	FJ391177		
SEMIA 6319		FJ390978	FJ391018	FJ391058	FJ391178		
SEMIA 6374		FJ390979	FJ391019	FJ391059	FJ391179		
SEMIA 6434		FJ390980	FJ391020	FJ391060	FJ391180		
SEMIA 6440		FJ390981	FJ391021	FJ391061	FJ391181		treeclusta Iomega
SEMIA 656		FJ390946	FJ390986	FJ391026	FJ391146	HQ634882	HQ634901
SEMIA 695		FJ390947	FJ390987	FJ391027	FJ391147		
SEMIA 928		FJ390948	FJ390988	FJ391028	FJ391148		
*Rhizobium pisi *strain DSM 30132		EF113149.1	JQ795193.1	JN580715.1	EF113134.1	JQ795183.1	JQ795190.1

**Figure 1:**
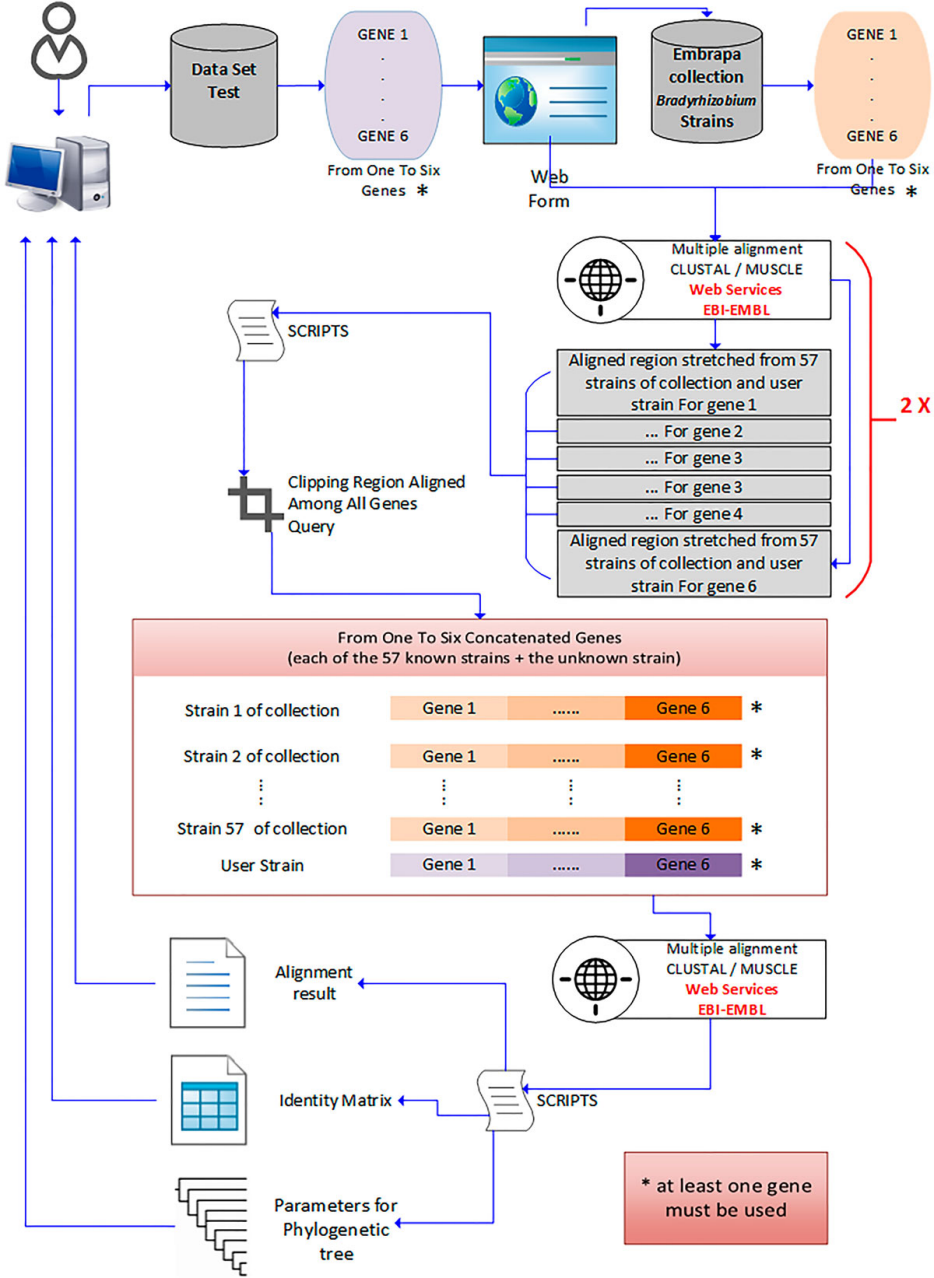
**Workflow for the taxonomic identification of *Bradyrhizobium *genus**.

All genes chosen in our work were verified for the MLSA requirements stated previously [13,25,27,36-38]. Our main goal is to allow, in a web environment, the search, analysis and phylogenetic inferences of the genus *Bradyrhizobium*. An overview of the steps and how they are interconnected is shown in Figure 1.

Observing Figure 1, we see that the user must provide data from one to six genes in the analysis. The next step consists of loading of the sequences stored in the database according to the sequences of the genes inserted by the user. Thus, the multiple alignment is performed by considering the input and the database sequences through the EBI-EML web service from which the user can choose to use the CLUSTAL Omega or MUSCLE algorithms. After performing the multiple alignment, a script will select and cut off the aligned regions of all sequences related to each specific gene. This task will produce sequences of equal sizes. After the alignment of all sequences for each one of the three genes, a new script will perform a concatenation of the gene sequences, thus producing a new sequence. At the end of this process, a new multiple alignment is performed with the concatenated sequences, and the results are processed by a script in order to produce the following outputs:

• Similarity Matrix/*score;*

• Text with the results of the multiple gene alignments;

• Parameters for phylogenetic tree generating;

which will assist in the classification of the organism.

The similarity matrix (score) produces an objective result, from which it is possible to verify the proximity between sequenced species (input) and all species available in the *Bradyrhizobium *database containing the three selected genes by the user.

## Utility and Discussion

In our study, validation was performed by using 16 strains, 14 of which represent type strains of the genus *Bradyrhizobium: B. betae *LMG 21987*^T^*, *B. canariense *LMG 22265*^T^*, B. *cytsi CTAW*11*^T^*, *B. diazoefficiens *SEMI A 5060, *B. diazoefficiens *SEMIA 5080, *B. diazoefficiens *SEMIA 6059, *B. diazoefficiens *USDA 110*^T^*, *B. elkanii *USDA 76*^T^, B. iriomotense EK*05*^T^, B. japonicum *USDA 6*^T^*, *B. japonicum *SEMIA 5079, *B. jicamae *PAC 68*^T^*, *B. lablabi *CCBAU 23086*^T^*, *B. lianingense *LMG 18230*T*. A sequence representing an outgroup was included in the database: *Rhodopseudomonas palustris *CGA009. The last adopted sequence belongs to *R. pisi *DSM 30132T, included as a negative control, i.e. a strain belonging to the genus *Rhizobium *rather than *Bradyrhizobium*. All genome sequences were collected from GenBank [43].

As presented in Sec. "Multilocus Sequence Analysis (MLSA)", the analysis of multiple genes in bacterial taxonomy consists of the joint sequencing (one concatenated sequence) analysis of housekeeping genes, and it has been proposed that, initially, at least five genes should be analysed [21,24,38]. For the MLSA methodology in this study, we proposed the use from one to six housekeeping genes, based on results obtained in recent studies, that similar results were obtained with three and with five genes [14,39-41,46-49]. However, as mentioned before, our site allows the analysis from one to six genes. The genes chosen as an input test were combined in three subgroups: (atpD, dnaK, glnll), (atpD, dnaK, glnII, recA) and (dnaK, recA, gyrB). Table 2 shows how the subsets of tests were assembled. Although it is present in the database, the rpoB gene was not used in the test because there were no available sequences for 29 strains. The default values to perform the alignment algorithms can be observed in Table 3. It has been generally accepted that strains with 16S rRNA gene similarities higher than 97.00% belong to the same species [23,50], but later, with the analyses of several 16S rRNA gene sequences, [51] proposed a cut-off value of 98.70-99.00%. However, when genes other than those for 16S rRNA gene are considered, lower values can be accepted. For example, [52] proposed an average nucleotide identity (ANI) value of 96.00%. However, for this study, we were strict, and for the tests, we assumed an initial cut-off of 98.70% in the MLSA analysis.

**Table 2:** Subset of genes used to test the proposed database by the MLSA methodology.

Quantity of Strains	Quantity of Strains for Genes Used	Quantity Genes	Algorithm for the Multiple Sequence Alignment	Genes Used
16	57	3	CLUSTAL Omega	atpD, dnaK, glnll
16	30	3	CLUSTAL Omega	dnaK, recA, gyrB
16	57	4	CLUSTAL Omega	atpD, dnaK, glnll, recA
16	57	3	MUSCLE	atpD, dnaK, glnll
16	30	3	MUSCLE	dnaK, recA, gyrB
16	57	4	MUSCLE	atpD, dnaK, glnll, recA

**Table 3:** Parameters for the execution of multiple sequence alignment algorithm.

Algorithm	Parameter	Value	Algorithm	Parameter	Value
CLUSTAL Omega	Sequence type	DNA	MUSCLE	Output format	Pearson/Fasta
CLUSTAL Omega	Output format	Pearson/Fasta	MUSCLE	Output tree	none
CLUSTAL Omega	Dealing input sequences	false	MUSCLE	Output order	aligned
CLUSTAL Omega	Mbed-like clustering guide-tree	true			
CLUSTAL Omega	Mbed-like clustering iteration	true			
CLUSTAL Omega	Number of combined iterations	0			
CLUSTAL Omega	Max guide tree iterations	-1			
CLUSTAL Omega	Max hmm iterations	-1			
CLUSTAL Omega	Order	aligned			

The table available as additional file 2 shows an identity matrix created where the values represent the similarity values between the sequences of the species of the database and the species used for the input test, *Bradyrhizobium *betae LMG 21987*^t^*. AS expected, the similarity rate of 100% was found between the input test and the species *B. betae *LMG 21987*^T^*. The similarity matrix also allows confirms the current taxonomy of the *Bradyrhizobium *genus (5), with *B. betae *LMG 21987*^T ^*showing higher similarity with *B. diazoefficiens *strains SEMIA 5060, SEMIA 5080, SEMIA 6059 and with the type strain *B. diazoefficiens *USDA 110*^T^*, of 96.29%, 96.13%, 96.06% and 96.06, respectively. None of the three strains was found to be the same species as the input test because they are all below the cut-off of 98.70%.

The table available as additional file 3 shows the values for the accuracy, precision, recall and f-score achieved with the software and strains available in the proposed database, these measures were calculated using data from the result of Matrix Identity generated by the analysis of multiple genes, with the use of the proposed cut-off of 98.70% for minimum similarity. The data sets used for the tests are described in additional file 3 and represent how the genomic sequences were grouped for analysis of multiple genes, along with the chosen implementation for multiple alignment algorithm. The data sets SI, S2 and S3 were analysed by the algorithm CLUSTAL OMEGA, and the combinations of the genes for these assemblies were arranged as (ATPD+DnaK+glnll), (dnaK+recA+gyrB) and (atpD+DnaK+glnll+recA) respectively. In the case of data sets S4, S5 and S6, the selected genes were the same as previous data sets, including maintaining the order, however, the analysis was performed using the MUSCLE algorithm. Each of the sequences was tested six times taking into consideration the parameters described above, and the results are shown in Tables 4 and additional file 3. The different subsets of genes resulted in differences in the results of the multiple alignments.

**Table 4:** Summary of the results.

Algorithm	Genes	Analysed Organisms	Cut Off Used	True Positive	False Positive	True Negative	False Negative
Muscle	atpD dnaK glnll recA	57	98.70%	33	0	853	26
Clustal Omega	atpD dnaK glnll recA	57	98.70%	30	0	857	25
Clustal Omega	dnaK recA gyrB	30	98.70%	27	0	445	8
Muscle	atpD dnaK glnll	57	98.70%	26	6	847	33
Muscle	dnaK recA gyrB	30	98.70%	25	0	445	10
Clustal Omega	atpD dnaK glnll	57	98.70%	24	0	853	35

Using algorithm CLUSTAL Omega the subset of genes atpD+dnaK+glnll shows values of 96.16% for accuracy, 100.00% for precision, 65.83% for recall and 73.64% for f-score, while considering the subset of genes dnaK+recA+gyrB, the values were of 98.33%, 100.00%, 85.78% and 88.89%, for subset with 4 genes atpD+dnaK+glnll, the values were of 97.26%, 100.00%, 75.39% and 81.39% for accuracy, precision, recall and f-score, respectively.

Using MUSCLE algorithm for analyse the same subset of genes atpD+dnaK+glnll shows values of 95.72% for accuracy, 92.00% for precision, 66.94% for recall and 71.26% for f-score, while considering the subset of genes dnaK+recA+gyrB, the values were of 97.92%, 100.00%, 82.44% and 86.98%, and for subset with 4 genes atpD+dnaK+glnll, the values were of 97.15%, 100.00%, 77.50% and 82.59% for accuracy, precision, recall and f-score, respectively.

Using the CLUSTAL Omega algorithm and the dnaK+recA+gyrB genes, the strain *B. diazofficiens *SEMIA 5080 was correctly identified as *B. diazoefficiens; *the classification indicated similarities of 99.92% with strain SEMIA 5060, of 99.52% with SEMIA 6059 and of 99.20% with the type strain *B. diazoefficiens *USDA 110*^T^*. This result indicates the correctness of the method for the classification of these SEMIA strains, which are different but fit into the same *B. diazoefficiens *species. The genes atpD+dnak+glnll analysed with the same algorithm showed similarities of 99.84% with *B. diazoefficiens *SEMIA 5060, 99.59% with *B. diazoefficiens *USDA 110*^T ^*and 85.28% for *B. diazoefficiens *6059.

In an additional test, considering the sequences related to *B. japonicum *strain SEMIA 5079 as input, we found that genes dnaK+atpD+glnll analysed with the CLUSTAL algorithm Omega resulted in the correct identification of the species and that the strain showed similarity with other strains, of 99.69% with *B. japonicum *USDA 6*^T ^*and of 98.84% with SEMIA 511. When analysed with the MUSCLE algorithm, the results were of 99.69% with *B. japonicum *UADA 6*^T^*, of 99.30% with SEMIA 512 and of 98.83% with SEMIA 511.

Another result demonstrating increased precision from the selection of certain genes was observed in the analysis of the species B. liaoningense LMG 18230*^T^*. When atpD+dnaK+glnll+recA genes were chosen, the algorithm CLUSTAL Omega presented a similarity of 97.60% between the type strain with the strain SEMIA 5025, while Muscle algorithm shows a 97.50% of similarity, whereas the analysis of atpD+dnaK+glnll genes resulted in a similarity of 97.17% using the Omega CLUSTAL and of 97.20% using the MUSCLE algorithm.

When the test set was used with genomic sequences of the species Rhizobium pisi, the classification resulted in values ranging from 30.00% to 82.15%, considering all the combinations involving alignment algorithms and subsets of genes. The results indicate the correct classification of *Rhizobium *pisi as not belonging to a species of *Bradyrhizobium *as described in additional file 3.

**Figure 2:**
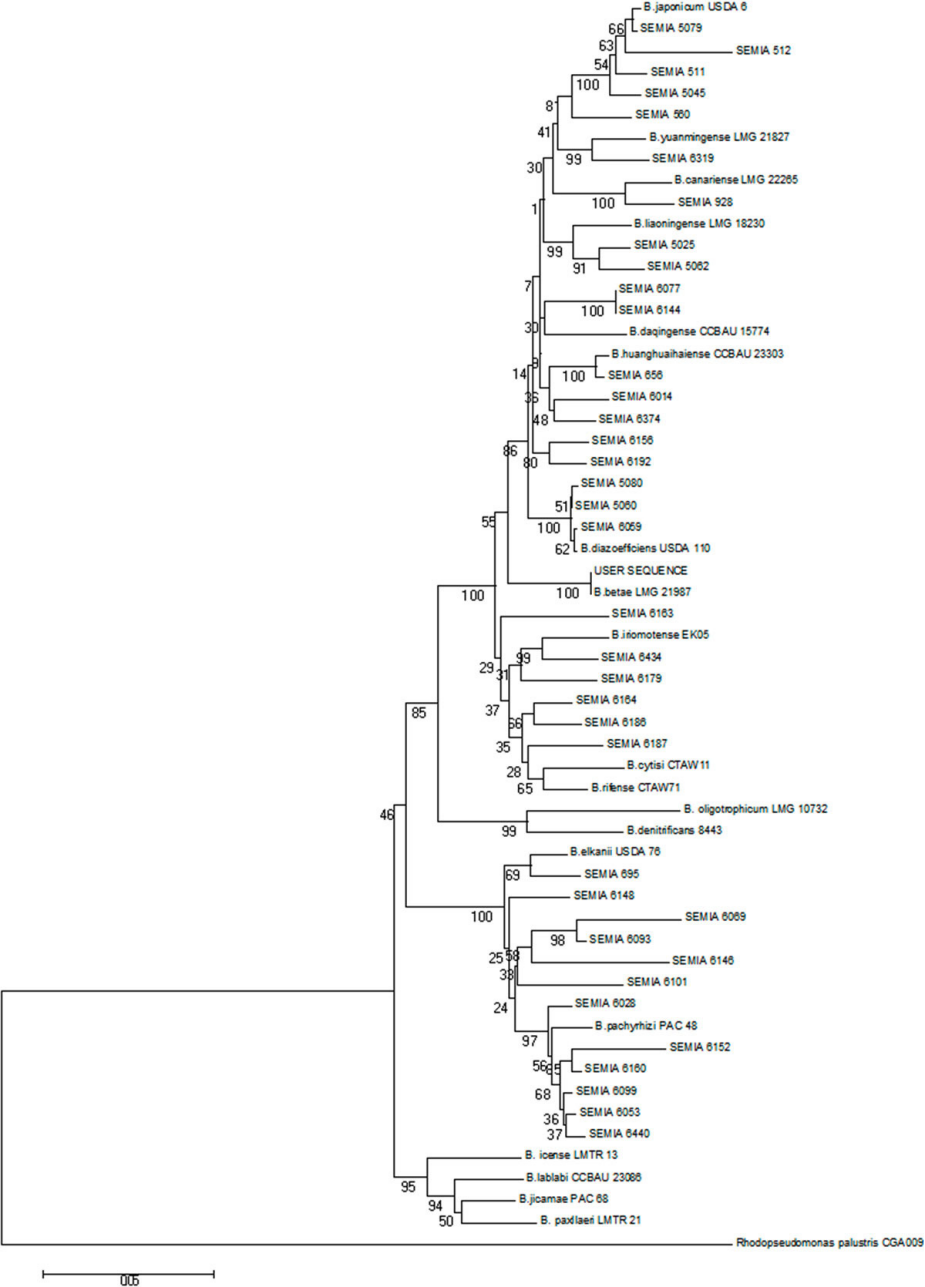
**Phylogenetic tree created from the results of three genes concatenated by the proposed methodology, strain of test based in *B. betae *LMG 21987, the evolutionary history was inferred using the Neighbour-Joining **[55]. The percentage of replicate trees in which the associated taxa clustered together in the bootstrap test (1000 replicates) are shown next to the branches [56]. The evolutionary distances were computed using the Tamura-Nei method [57] and are in the units of the number of base substitutions per site. The analysis involved 25 nucleotide sequences. All positions containing gaps and missing data were eliminated. There were a total of 1152 positions in the final dataset. Evolutionary analyses were conducted in MEGA6 [54].

Figure 1 shows the outputs for taxonomic and phylogenetic identification available in the proposed database. The identification of the genus *Bradyrhizobium *through MLSA also brings the results of multiple alignment and parameters for creating phylogenetic trees, both of which have bearing on the phylogenetic implications regarding the organisms of interest [53]. The alignment of a single sequence obtained from the concatenation of three genes produced by the application of the MLSA methodology was used to better explain how the phylogenetic tree can be inferred from the database analysis. A phylogenetic tree was produced with Mega software version 6 [54] shows in Figure 2, by considering the previous results shown in Table available as additional file 2. In this figure, it is possible to verify the correct classification of the test species, as well as the species *B. betae *LMG 21987*^T ^*with 100% similarity.

## Conclusion

This work was developed in order to provide a database for the taxonomic and phylogenetic identification of the genus *Bradyrhizobium *by using the multilocus sequence analysis (MLSA) methodology. More specifically, the following tools and database functionality were developed:

• a database based on a relational model using BioSQL to store data and to maintain the interoperability between bioinformatics projects such as BioPerl, BioPython and BioJava;

• a database with validated information of Bradyrhizobium species through a friendly web interface for users;

• computational tools suitable for the automatic data mining, analysis and classification of genomic sequences;

• computational scripts for the automatic updating of the database with sequences used in the identification and classification process;

The experimental results indicate that the proposed database and the computational tools correctly distinguished species of the same genus and with high similarity rates, reinforcing the efficiency of the MLSA methodology. The Results also show that for the efficient use of the MLSA database it is important to know the combinations of genes that will be used in the taxonomic analysis, as well as the similarity rates that could be used for each genus. Therefore, it is necessary to perform previous tests in order to achieve the best results. The proposed database provides useful information for research in taxonomy and molecular phylogeny of the genus *Bradyrhizobium*, taking into account the possibility of gathering into a single database information that is commonly needed for studies of these microorganisms and is fragmented in various sources and formats. The current database contains 286 entries of gene sequences of the *Bradyrhizobium *genus. However, further studies are planned to include sequences of other rhizobial genera: *Rhizobium, Sinorhizobium, Azorhizobium, Mesorhizobium *and *Neorhizobium*. There is also the possibility of increasing the number of genes to be analysed. Finally, it is important to integrate the current results with other software packages that allow the visualization of the results directly into a web page, creating an association that will make it even more simple and practical to interpret phylogenetic implications from the proposed database.

## Competing interests

The authors declare that they have no competing interests.

## Authors' contributions

HA conceived the idea, assembled the datasets, performed the analysis, developed the computational method and contributed to drafted the manuscript; FML conceived the idea, developed the computational method and drafted the manuscript; PRS helped in the assembled the datasets and developed the computational method; MH conceived the idea, contributed to the analysis of results and helped to draft the manuscript.

## Supplementary Material

Additional file 1 **The adopted BioSQL relational model**. Click here for file

Additional file 2 **Identity matrix created where the values represent the similarity values between the sequences of the species of the database and the species used for the input test**. Identity matrix generated after performing the taxonomic analysis available in the proposed database, using CLUSTAL Omega algorithm and the subset of genes atpD, dnaK, glnll; User Sequence (l); *B.canariense *LMG 22265*^T ^*(2); *B. liaoningense *LMG 18230*^T ^*(3); *B. elkanii *76*^T ^*(4); *B. yuanmingense *CCBAU 21827*^T ^*(5); *B. japonicum *USDA6*^T ^*(6); *B. iriomotense *EK05*^T ^*(7); *B. pachyrhizi *48*^T ^*(8); *B. jicamae *68*^T ^*(9); *B. betae *LMG 21987*^T ^*(10); SEMIA 5079(11); SEMIA 5080(12); SEMIA 6059(13); *B. cytisi CTAW *11*^T ^*(14); *B. rifense CTAW *71*^T^*(15); *B. daqingense *CCBAU 15774*^T ^*(16); *B. lablabi *CCBAU 23086*^T ^*(17); *B. huanghuaihaiense *CCBAU 23303*^T ^*(18); SEMIA 5060 (19); *B. diazoefficiens *USDA 110(20); *R. palustris *CGA009(21); *B. oligotrophicum *LMG(22); *B. paxllaeri *LMTR 21(23); *B. icense *LMTR 13(24); *B. denitrificans *LMG 8443(25); SEMIA 511(26); SEMIA 512(27); SEMIA 560(28); SEMIA 656(29); SEMIA 695(30); SEMIA 928(31); SEMIA 5025(32); SEMIA 5045(33); SEMIA 5062(34); SEMIA 6014(35); SEMIA 6028(36); SEMIA 6053(37); SEMIA 6069(38); SEMIA 6077(39); SEMIA 6093(40); SEMIA 6099(41); SEMIA 6101(42); SEMIA 6146(43); SEMIA 6148(44); SEMIA 6152(45); SEMIA 6156(46); SEMIA 6160(47); SEMIA 6163(48); SEMIA 6164(49); SEMIA 6179(50); SEMIA 6186(51); SEMIA 6187(52); SEMIA 6192(53); SEMIA 6319(54); SEMIA 6374(55); SEMIA 6434(56); SEMIA 6440(57); SEMIA 6144(58) Click here for file

Additional file 3 **Average classical measures of classification for 16 strains used as input sequences in tests**. This table shows the values for the accuracy, precision, recall and f-score achieved with the software and strains available in the proposed database, these measures were calculated using data from the result of Matrix Identity generated by the analysis of multiple genes, with the use of the proposed cut-off of 98.70% for minimum similarity. Click here for file

## Declarations

The authors declare that funding for publication of the article was sponsored by UTFPR - Federal University of Technology - Paraná and CNPq grant # 562008/2010-1.

This article has been published as part of *BMC Genomics *Volume 16 Supplement 5, 2015: Proceedings of the 10th International Conference of the Brazilian Association for Bioinformatics and Computational Biology (X-Meeting 2014). The full contents of the supplement are available online at http://www.biomedcentral.com/bmcgenomics/supplements/16/S5.
