# Supplementary figures and images for: A database for the taxonomic and phylogenetic identification of the genus Bradyrhizobium using multilocus sequence analysis
Source: BMC Genomics. 2015 May 26;16(Suppl 5):S10. doi: 10.1186/1471-2164-16-S5-S10 (PMC4460661; doi:10.1186/1471-2164-16-S5-S10)

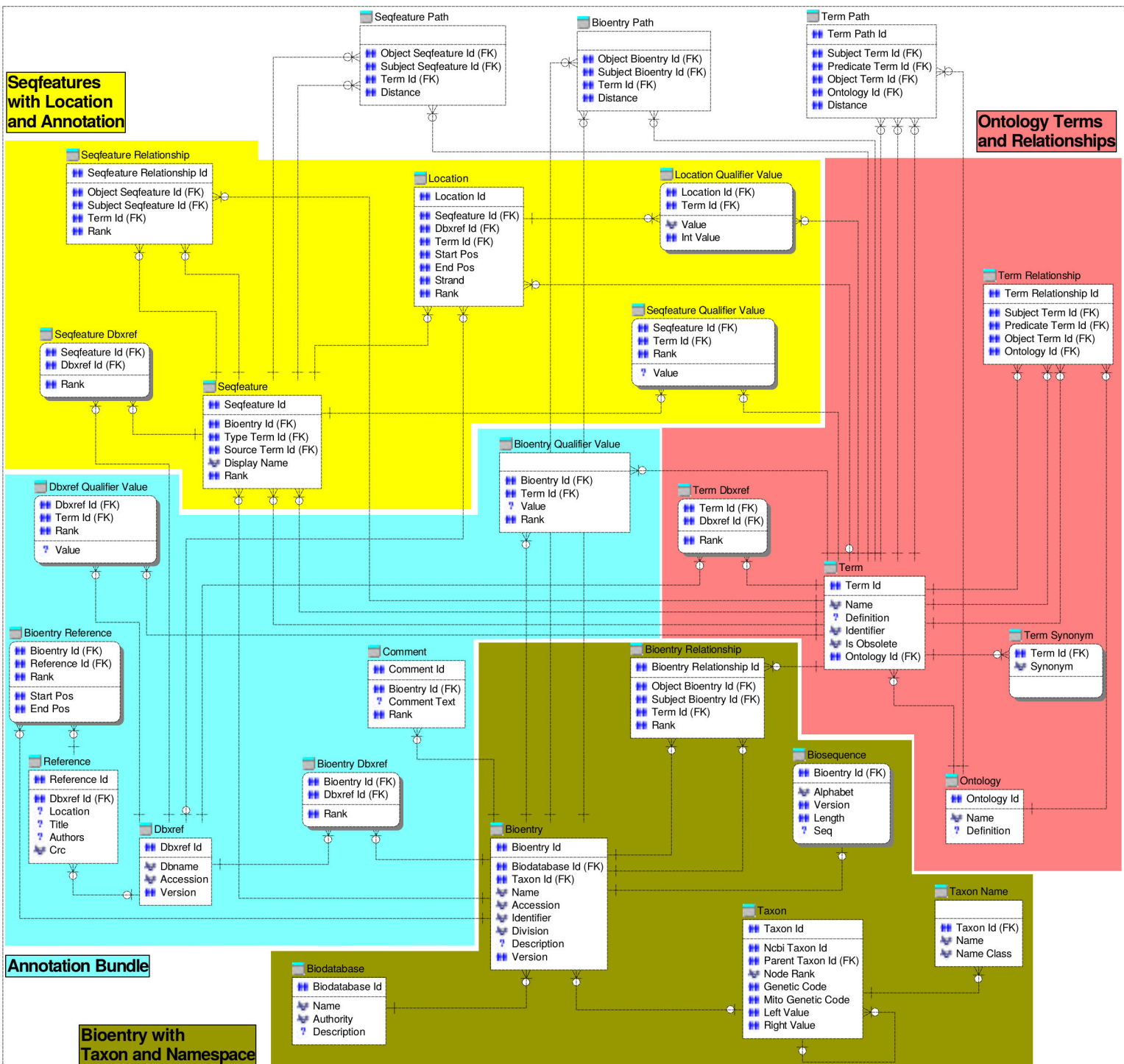

Supplement: Additional file 1 — The adopted BioSQL relational model. [file 1471-2164-16-S5-S10-S1.pdf]
